# Ratat1: A Digital Rat Brain Stereotaxic Atlas Derived from High-Resolution MRI Images Scanned in Three Dimensions

**DOI:** 10.3389/fnsys.2016.00064

**Published:** 2016-08-04

**Authors:** Kurt Wisner, Boris Odintsov, Daniel Brozoski, Thomas J. Brozoski

**Affiliations:** ^1^Auditory Research Group, Division of Otolaryngology – Head and Neck Surgery, Southern Illinois University School of Medicine, SpringfieldIL, USA; ^2^Biomedical Imaging Center, Beckman Institute for Advanced Science and Technology, University of Illinois at Urbana–Champaign, UrbanaIL, USA

**Keywords:** rat brain atlas, high-resolution MRI, Long Evans rats, skull landmark indexed, coronal sagittal horizontal views

## Introduction

Numerous magnetic resonance images (MRI)-derived brain atlases are available online for multiple species (Bakker et al., 2015). MRI-derived rat brain atlases have been produced for different purposes using different procedures. The largest number of atlases appear to have been derived for morphometric purposes, e.g., white matter – gray matter segmentation (Valdes-Hernandez et al., 2011), volumetric analysis of major structures (Papp et al., 2014), developmental dynamics (Calabrese et al., 2013). Published rat atlases have drawn their data primarily from albino strains, i.e., Wistar or Sprague Dawley. The present MRI atlas, to our knowledge, is the first available for adult Long-Evans rats, a pigmented strain widely used in behavioral and perceptual research. Some atlases have taken a nomothetic approach, that is to say deriving average templates from groups of animals, e.g. (Valdes-Hernandez et al., 2011) while others have taken an idiographic approach, using data derived from a single exemplar, e.g. (Papp et al., 2014). The nomothetic approach attempts to portray features of a population-average brain, but does so at the risk of statistically blurring small or variable features. The idiographic approach, when combined with high resolution scans, captures small features without statistical blurring, but does so at the risk of including anomalous features. The present atlas was derived from two selected exemplars, thus taking an idiographic approach. In addition, a number of atlases, e.g. (Papp et al., 2014), link or index their images to the widely used reference system established by Paxinos and Watson (1998) (hereafter P&W). This enables users to take advantage of the superior structural detail provided by stained histological sections, and to adopt, if they wish, the widely used P&W coordinate system. An early rat MRI atlas, no longer available online (Schweinhardt et al., 2003), analytically morphed their MRI images to P&W image space using an affine transformation anchored to prominent brain landmarks. From their published examples, line drawings derived from the transformed images were portrayed on a P&W coordinate grid (e.g., **Figure 2**), but the actual MRI images were not (e.g., **Figure 3**) (Schweinhardt et al., 2003). The present atlas did not affine transform the MR images to P&W image space, but rather directly imaged P&W skull landmarks, Bregma and Lambda (reference points visible to a surgeon) and used these to anchor the MR images to the P&W coordinate system via a superimposed grid. The Papp et al. (2014) atlas imaged Bregma and Lambda, but did not project either the reference points or coordinates onto individual brain scans.

Animal MRI atlases vary considerably in their method of subject preparation, as well as acquisition parameters affecting image quality, e.g., magnet field strength. Images have been obtained from live subjects (Valdes-Hernandez et al., 2011), *ex vivo* subjects (as in the present atlas), and preserved tissue (Papp et al., 2014). Each method has advantages and disadvantages. *In vivo* acquisition obviously captures the brain in its native state, however, movement artifact may affect image quality and anesthesia tolerance limits total acquisition time (which also limits image resolution). Images obtained from preserved tissue eliminate the issues of movement artifact and acquisition time but bring with it structural changes from preservative perfusion. The *ex vivo* method, used for the present atlas, maintains *in situ* structure, but acquisition time is constrained by post-mortem tissue degradation (approximately 10 h in the present environment). Higher magnet field strength brings with it improved spatial resolution, but also imaging artifacts that accrue in proportion to field strength (discussed below).

## Objective

To derive a high resolution digital brain atlas of *in situ* MR images from adult Long Evans rats suitable for use in stereotaxic surgery applications. Using an ultra-high resolution scanner, brain images were acquired *ex vivo* in a near natural state. Those images, particularly when combined with images derived from conventional histological sections, can be used to determine the coordinates of stereotaxic surgical targets with improved accuracy. Additional objectives were to depict brain images in three viewing planes indexed to the skull surface as well as to the skull landmarks Bregma and Lambda. A further objective was to present the atlas in a simple widely used file format (.pdf) not requiring specialized software for viewing, copying, or unpacking.

## Subjects

Nine adult Long Evans (Harlan, Indianapolis, IN, USA) male rats, wt. 350–550 g were imaged. The image sets of two animals were judged to be of superior quality and were used as exemplars to compose the atlas. Their images were free of anatomical anomalies and imaging distortions. These animals were 135–136 days of age when imaged, and weighed 506 and 510 g. The experimental protocol was approved by the Laboratory Animal Care and Use Committee of Southern Illinois University School of Medicine (protocol #149-06-026).

## Imaging

The animals were imaged under identical conditions on successive days. MRI scans were acquired on a ultra-high resolution vertical bore (89 mm) micro-imaging scanner (Oxford Instruments, Abington, UK) equipped with a Unity/Inova console (Varian, Palo Alto, CA, USA), operating at 14.1 T. A high image quality spin-echo-multi-slice (SEMS) protocol was employed. A long repetition time (TR = 4.0 s) and short echo-time (TE = 9.0 ms) were used to provide the best anatomical image contrast. Images were acquired slice by slice with no gap between slices. Receiver gain and radiofrequency (RF) transmit power were optimized utilizing VnmrJ prescan options. Image slices were 0.2 mm thick, with a planar resolution of 50 μm, and were acquired using 20 scans per slice. Images were processed using custom-written Matlab codes that used signal-to-noise (SN) normalization to reduce contrast variation between slices and improve image quality. Commercial (Varian/Agilent) Vnmrj software normalizes the SN of each slice individually to improve their visual appearance. This alteration, however, makes it difficult to quantitatively compare slices. To reduce artificial contrast variation between slices, the custom-written Matlab codes that were used, normalized slices to the average SN of the image set.

A patented tunable transmit/receive RF coil was used for image acquisition (Odintsov, 2011). For each brain: 120 transverse (i.e., coronal) slices were obtained, extending from the rostral frontal cortex through the caudal brainstem; 75 sagittal slices were obtained, extending from left to right temporal cortical surfaces, of which 70 were used in the atlas; 75 horizontal slices were obtained, extending ventrally from the dorsal cortical surface, of which 48 were used in the atlas.

## Subject Preparation

Scans were acquired *in situ* and *ex vivo*. *Ex vivo* imaging eliminated movement artifacts and permitted the long acquisition times necessary to optimize image resolution. This preparation also facilitated Bregma and Lambda marking as described below. Immediately before imaging, the rats were lethally anesthetized (Euthasol, Virbac, Fort Worth, TX), decapitated, and their dorsal skull exposed. Under visual inspection, 1 mm holes were drilled into the skull at Bregma and Lambda (as defined in P&W). Each hole was filled with Gelfoam^®^ saturated with 3 mM CuSO_4_. The CuSO_4_ provided an empirical image phantom at Bregma and Lambda the centroid of which was easily determined later by viewing the set of images containing the marker. The head was placed in a polyethylene holder along with a laterally positioned glass capillary also filled with CuSO_4_. The image phantom of the capillary indexed the left hemisphere, making image laterality unambiguous. Images from Rat 1 were used for transverse slices, while images from Rat 2 were used for sagittal and horizontal slices. Selection of representative animals was based on the conjoint criteria of (a) the brain being free of anatomical anomalies (e.g., tumors, infarcts), (b) good bilateral symmetry and accurate alignment in the imaging field of view, and (c) freedom from imaging artifacts (e.g., warping, shadowed areas).

## Image Depiction and Refinement

**FIGURE 1:**
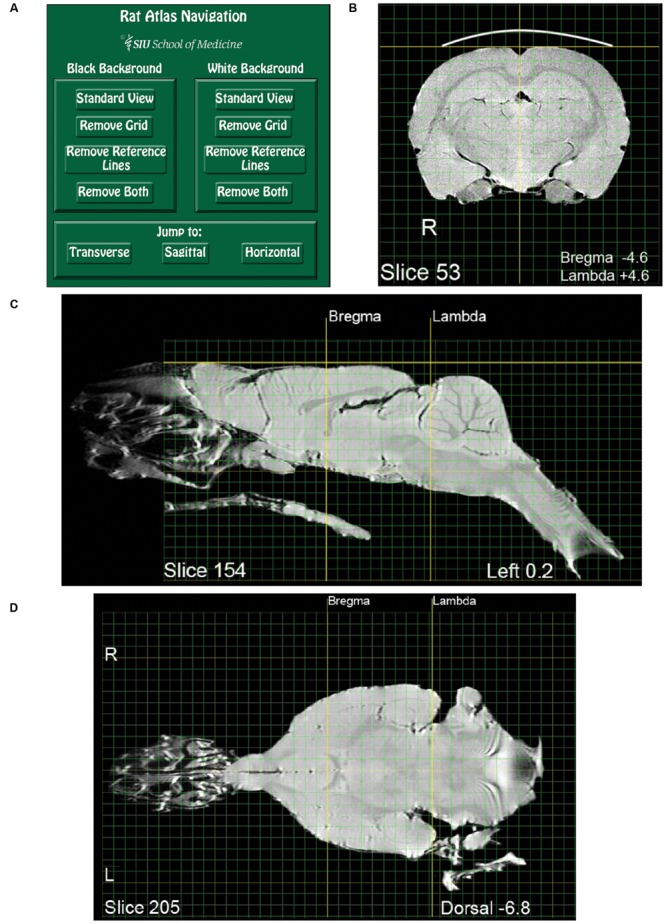
**Pages from the magnetic resonance images (MRI) rat atlas. (A)** Depiction of the atlas home page with control buttons for navigation and selection of display settings. **(B)** An exemplary transverse (i.e., coronal) slice located midway between Bregma and Lambda. **(C)** An exemplary sagittal slice located 0.2 mm left-lateral of midline. Note that the olfactory epithelium and the bony and soft palate are included in the image. **(D)** An exemplary horizontal slice located 6.8 mm ventral to the dorsal-most surface of the brain [indicated by the gold horizontal reference line in **(C)**].

JPEG images of each brain slice, produced by MatLab code, were imported into Adobe Photoshop CS2 v9.0 (Adobe Systems^1^). The images were saved in Photoshop document format at 500 pixels/inch and Photoshop was used to modify selected aspects of each image. The images were rotated to a normal orientation and placed on a 1 mm grid. The grid was aligned rostro-caudally and laterally with Bregma, and dorso-ventrally with the dorsal-most surface of the brain. Image dimensions were unaltered. Descriptive labels were added to each slice and tissue surrounding the brain was digitally removed, with important exceptions. Some brain sections retain images of external tissue of interest, such as the middle and inner ear (**Figure 3**) and olfactory epithelium (**Figures 1C,D**). A bold line depicting the dorsal skull was added to each transverse image. This skull indicator was superimposed over the faint image of the skull. Within each image set (transverse, sagittal, or horizontal) one slice was chosen as a “standard,” exhibiting visually determined optimal levels of contrast and brightness with respect to other images in the set. Visual inspection was used to optimize the brightness and contrast of individual images in each set so as to approximate that of the standard. Horizontal slices required an additional adjustment to bring them more precisely into a plane perpendicular to the sagittal plane of the brain. To achieve this, hemi-sections from adjacent horizontal slices displaced by 0.4 mm were cut and pasted to achieve a 90° horizontal orientation. Examples of the three planar views are shown in **Figures 1B–D.** Images comprising the atlas were derived from the individual subject scans as described above, without averaging across subjects. After refinement the images were exported from Photoshop in PDF format and assembled into a PDF book, one image to a page.

## Use: Interactive Features

Note that when using the uncompressed version of the atlas available from figshare.com, https://figshare.com/articles/RatAtlas_2_0_Locked_pdf/3144955 the atlas must be downloaded to the user’s computer and opened using Adobe Acrobat or Adobe Reader in order to use the atlas’ interactive features. While the atlas interactive features may be functional using file readers other than Adobe, this has not been tested by the authors.

## Use: Viewing the Atlas

Adobe Reader or Acrobat (Adobe Systems Inc.^2^) can be used for viewing the atlas, as well as for layer manipulation, navigation, and copying. Other applications that open PDF files might work as well, but were not verified. A compressed version of the atlas is appended as a supplement, while an uncompressed version is available at https://figshare.com/articles/RatAtlas_2_0_Locked_pdf/3144955. The first page of the atlas is a navigation page used to select viewing options (**Figure 1A**). In the default state, all layers comprising each slice are visible (Brain, Grid, Reference Lines, and Black Background). Clicking the “White Background” button on the navigation page will turn off the dark background layer on all slices creating a view useful for printing or copying into other documents. Clicking “Black Background” will restore all default settings. Additional buttons are available to toggle on and off the grid and reference lines with either the black or white background. Users who wish to use their own applications for reconstruction or analysis may want to choose the background clearing options before exporting images. The three buttons at the bottom of the navigation page enable jumping to the first slice of either the transverse, sagittal, or horizontal image sets. Aside from using the navigation page, the layers comprising each slice can be switched on and off directly from the slice viewing pane. This is done using the “Layers” tab located in the Adobe navigation pane.

## Use: Explanation of Atlas Layers

Each slice depiction appears on a separate page and consists of four layers that can be independently turned on or off as described above. *Brain* depicts the brain and its associated labels (Bregma/Lambda coordinates, Slice #, etc.). *Grid* depicts the rectilinear gridlines. Green lines indicate 1 mm intervals and yellow lines 1 cm intervals. The grid is aligned with Bregma and the dorsal-most surface of the brain. *Reference Lines*, shown in bold, index the dorsal surface of cortex, and midline in the transverse sections. For the sagittal and horizontal sections, the reference lines index Bregma and Lambda. Images can be viewed with (black) or without (white) a *Background*.

## Use: Zoom Control

Zooming is accomplished as in other PDF documents. The default zoom displays one full page, i.e., a slice. This setting can be restored using the Adobe view menu, highlighting zoom, and selecting “Zoom to page level” (Ctrl+0).

## Use: Copying

Once the atlas is open for viewing, e.g., using Adobe Reader, it can be saved (“Save As”) in its entirety to local media, such as a hard drive or flash memory. Individual sections can be copied from the Atlas as well. To do so open an online or local copy of the Atlas and navigate to the section of interest. With the section displayed on screen, left click “Edit” and “Take a Snapshot” from the drop-down menu. A single left click anywhere on the displayed image will place a copy of the image into a buffer. The copy can then be pasted into a user file such as a Word document, Photoshop image, or PowerPoint slide. Alternatively, instead of clicking on the image, a left click and drag can be used to highlight a sub-area of the image. In that case only the highlighted portion will be copied. To print a brain section from the Atlas, select (left click) the thumbnail of the image of interest, then right click. A pop-up print window enables printing.

## Use: Defining Structures and Applying Labels

**FIGURE 2:**
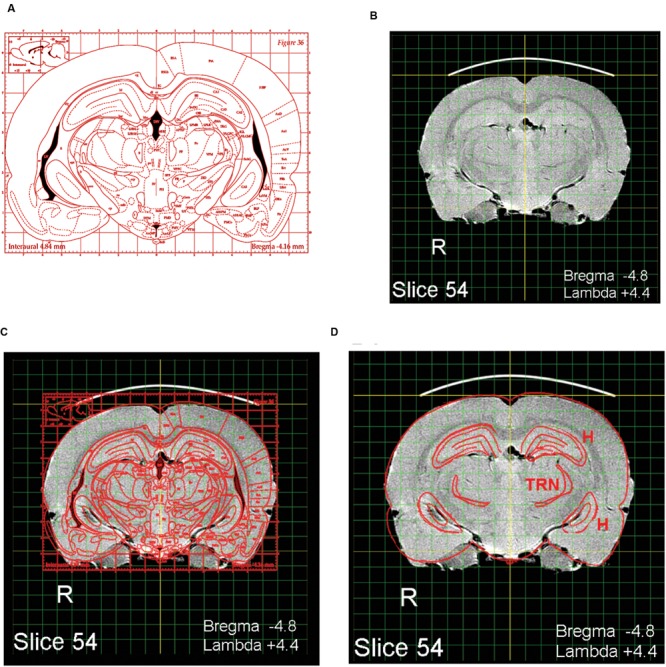
**Identifying areas of interest using image overlays. (A)** A labeled digital outline drawing taken from Paxinos and Watson (1998) containing two hypothetical areas of interest: The hippocampus (H) and thalamic reticular nucleus (TRN). The areas of interest were chosen to include one obvious structure (H) and one less obvious structure (TRN). **(B)** A slice taken from the MRI atlas with features matching the P&W section. **(C)** The P&W section superimposed on the MRI slice; dorso-ventral and lateral scale adjustments were made to A to align it with B. **(D)** Features not of interest have been erased.

**FIGURE 3:**
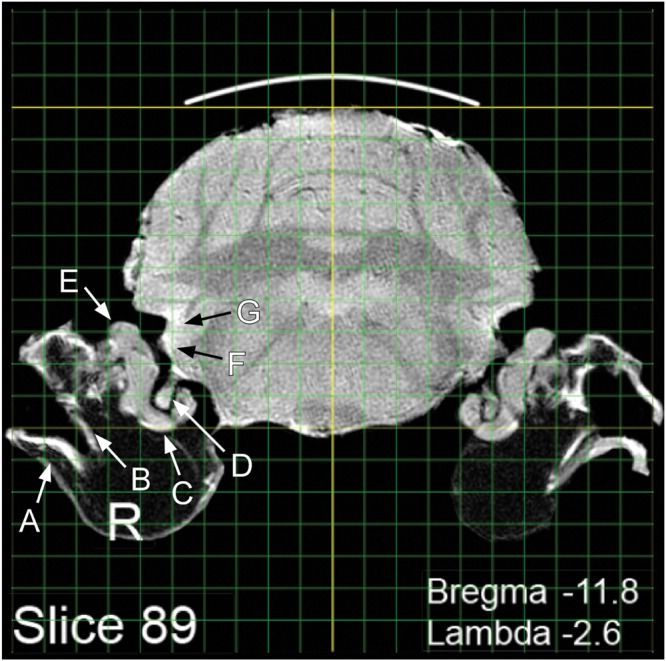
**Visualization of peripheral and central structures of the auditory/vestibular pathway: A, tympanic membrane; B, ossicular chain; C, cochlea; D, auditory nerve; E, vestibular organs; F, ventral cochlear nucleus; G, dorsal cochlear nucleus**.

The present MRI images depict the brain largely undistorted with respect to its *in vivo* state. While many structures are clearly visible, other structural boundaries are not as distinct as in stained histological sections. Identifying and labeling indistinct structures can be done by superimposing a digital atlas outline, such as the outline drawings of P&W onto an MRI image. An intuitive way to do this is to use the image layering and scaling applications in Photoshop. A composite overlay can be made by first selecting a P&W outline depicting areas of interest (**Figure 2A**). For purposes of exposition, a P&W drawing (“Figure 36”) containing an easy-to-visualize (hippocampus, H) and a difficult-to-visualize area (thalamic reticular nucleus, TRN) has been chosen. Bregma/Lambda numeric indices, plus obvious well-defined features such as the hippocampus and medial geniculate body, assist in selecting a matching MRI slice from the present atlas (**Figure 2B**). The P&W outline was then laterally and dorso-ventrally scaled to align with the MRI slice (**Figure 2C**). Scale matching is required not only because the P&W images were obtained from rats of a different strain and size, but also because P&W depictions were derived from photographs of histological sections subject to shrinkage in processing. Other distortions might also be present in P&W depictions. Compared to the MRI sections, which capture the brain close to its *in vivo* state, P&W sections appear slightly compressed along the dorso-ventral axis. The advantage of using a hybrid overlay, such as shown in **Figure 2D**, is improved accuracy, i.e., the MRI image has not been altered by histological preparation, and identity confirmation using the superior structural delineation provided by the histological overlay. Once a structure of interest has been identified using the overlay as a guide, the structure can then be visualized in the MRI slice without the overlay. For example, using Photoshop, the overlay can be toggled between visible and invisible. This strategy cues the user into subtle features of the MRI image that define the structure of interest, but that at first examination escaped notice. Users might opt to further enhance their copy of the MRI image containing the structure of interest by increasing contrast or drawing in a border. Using the MRI image to guide stereotaxic surgery should improve accuracy of target acquisition, particularly when using adult Long Evans rats. A second advantage of using the present MRI images is that peripheral structures of interest can be included and visualized in relation to brain areas of interest. An example is shown in **Figure 3** Any digital image application capable of importing, layering, and scaling PDF images could serve this purpose.

## Author Contributions

TB, contributed to implementation, data collection and analysis, and wrote the manuscript. BO, contributed to implementation, collection and analysis of the MRI data, and contributed to the manuscript. DB, contributed to inception, data analysis, and manuscript preparation. KW, was responsible for the final image analysis and presentation, and contributed to the manuscript preparation.

## Conflict of Interest Statement

The authors declare that the research was conducted in the absence of any commercial or financial relationships that could be construed as a potential conflict of interest.

## Footnotes

1 www.adobe.com

2 www.adobe.com
